# Comparison of ONT and CCS sequencing technologies on the polyploid genome of a medicinal plant showed that high error rate of ONT reads are not suitable for self-correction

**DOI:** 10.1186/s13020-022-00644-1

**Published:** 2022-08-09

**Authors:** Peng Zeng, Zunzhe Tian, Yuwei Han, Weixiong Zhang, Tinggan Zhou, Yingmei Peng, Hao Hu, Jing Cai

**Affiliations:** 1 State Key Laboratory of Quality Research in Chinese Medicine, Institute of Chinese Medical Sciences, University of Macau, Macau, China; 2 School of Ecology and Environment, Northwestern Polytechnical University, Xi’an, China

**Keywords:** ONT-based assembly, Allotetraploid, *Veratrum dahuricum*, Low-quality sequences, Homozygous variants

## Abstract

**Background:**

Many medicinal plants are known for their complex genomes with high ploidy, heterozygosity, and repetitive content which pose severe challenges for genome sequencing of those species. Long reads from Oxford nanopore sequencing technology (ONT) or Pacific Biosciences Single Molecule, Real-Time (SMRT) sequencing offer great advantages in de novo genome assembly, especially for complex genomes with high heterozygosity and repetitive content. Currently, multiple allotetraploid species have sequenced their genomes by long-read sequencing. However, we found that a considerable proportion of these genomes (7.9% on average, maximum 23.7%) could not be covered by NGS (Next Generation Sequencing) reads (uncovered region by NGS reads, UCR) suggesting the questionable and low-quality of those area or genomic areas that can’t be sequenced by NGS due to sequencing bias. The underlying causes of those UCR in the genome assembly and solutions to this problem have never been studied.

**Methods:**

In the study, we sequenced the tetraploid genome of *Veratrum dahuricum* (Turcz.) O. Loes (VDL), a Chinese medicinal plant, with ONT platform and assembled the genome with three strategies in parallel. We compared the qualities, coverage, and heterozygosity of the three ONT assemblies with another released assembly of the same individual using reads from PacBio circular consensus sequencing (CCS) technology, to explore the cause of the UCR.

**Results:**

By mapping the NGS reads against the three ONT assemblies and the CCS assembly, we found that the coverage of those ONT assemblies by NGS reads ranged from 49.15 to 76.31%, much smaller than that of the CCS assembly (99.53%). And alignment between ONT assemblies and CCS assembly showed that most UCR can be aligned with CCS assembly. So, we conclude that the UCRs in ONT assembly are low-quality sequences with a high error rate that can’t be aligned with short reads, rather than genomic regions that can’t be sequenced by NGS. Further comparison among the intermediate versions of ONT assemblies showed that the most probable origin of those errors is a combination of artificial errors introduced by “self-correction” and initial sequencing error in long reads. We also found that polishing the ONT assembly with CCS reads can correct those errors efficiently.

**Conclusions:**

Through analyzing genome features and reads alignment, we have found the causes for the high proportion of UCR in ONT assembly of VDL are sequencing errors and additional errors introduced by self-correction. The high error rates of ONT-raw reads make them not suitable for self-correction prior to allotetraploid genome assembly, as the self-correction will introduce artificial errors to > 5% of the UCR sequences. We suggest high-precision CCS reads be used to polish the assembly to correct those errors effectively for polyploid genomes.

**Supplementary Information:**

The online version contains supplementary material available at 10.1186/s13020-022-00644-1.

## Background

Many medicinal plants have complex genomes due to high ploidy, heterozygosity, and repetitive sequences. Those genomes are very challenging to sequence and assemble with the short-read second-generation sequencing. With the rapid development of third-generation sequence technologies, more and more plants were sequenced and assembled using two representative kinds of long-read sequencing technologies. For example, *Arabidopsis thaliana* Ler [1], *Ananas comosus* (L.) Merr. [2], *Zea mays *[3] and *Brassica oleracea* var. capitata [4] were sequenced by Pacific Biosciences Single Molecule, Real-Time (SMRT) sequencing platform. The other one was Oxford nanopore sequencing technology (ONT), which was used to assemble *Brassica rapa*, *Brassica oleracea*, *Musa schizocarpa*, and *Sorghum bicolor* [5, 6]. Compared to the short reads and sequencing bias of second-generation technology, third-generation technology has an incomparable advantage with its long read and randomness of sequencing, which helps to resolve the assembly problems in complex genomic regions [7, 8].

However, the high error rate (ranging from 10 to 20%) of long reads from both ONT and SMRT [9–11] makes it a challenge to de novo assemble genomes. The recently developed CCS (circular consensus sequencing)-mode of SMRT has read of much lower error rates but sacrifices the read length to some extent. Therefore, except for CCS reads, it is generally necessary to correct those long reads before assembly. At present, several long reads de novo assemblers will firstly perform self-correction for the long reads, such as Canu [12], MECAT [13], HGAP [14], NextDenovo [15], and NECAT [16]. Error correction algorithms are designed to identify and repair or delete sequencing errors based on hybrid or non-hybrid methods. The hybrid method uses short reads or contigs assembled with short reads to correct the long reads while the non-hybrid method uses the overlapping information between the long reads for self-correction [17]. No matter which error correction method is adopted, it involves aligning with the long reads, and the inaccurate alignment will result in errors in the correction. Compared with diploids, polyploid genomes are more challenging. Although the average DNA sequence difference between subgenomes in the allopolyploid genome ranged from 14.1 to 27.8% in two allopolyploid species sequenced recently which is sufficiently different for de novo genome assembly of allopolyploid genomes, the sequence difference at local gapless alignments is only ~ 5% [18], which is much smaller than the sequencing error rate of long reads. These genomic areas of those gapless alignments will be impossible to be accurately assembled with self-corrected long reads because there are more sequencing errors than the true sequence difference between homologous regions in the polyploid genome.

In this study, we compared the performance of two mainstream third-generation sequencing technologies in an allotetraploid medicinal plant, *Veratrum dahuricum* (Turcz.) O. Loes (VDL). VDL is known as Lilu in traditional Chinese medicine, and its extract has a variety of pharmacological activities, including hypotensive, anti-thrombosis, and anti-tumor functions [19]. We compared ONT-based assemblies with the released genome assembly [20] using CCS reads, then investigated the cause of the high percentage of UCR in ONT-based assemblies. We proposed the hypothesis that, in addition to the sequencing errors, the over-correction of the long reads may lead to errors that could not be mapped by original reads in allopolyploid assemble. Our findings provide guidance on the selection of sequencing strategies for other complex genomes of medicinal plants.

## Materials and methods

### Sample and sequencing data

We collected a Chinese herb *Veratrum dahuricum* (Turcz.) O. Loes (VDL) from its native habitats in Jilin Province, China. Genome DNA was sequenced on three sequencing platforms, Oxford nanopore sequencing technology (ONT) platform, circular consensus sequencing (CCS) of Pacbio platform, and Illumina NextSeq 500 platform, obtaining 192.49 Gb ONT long reads, 55.03 Gb CCS reads and 135.08 Gb NGS reads, respectively [20].

### Genome de novo assembly using ONT reads

De novo assembly was carried out using several assemblers. Nextdenovo [21] (minimap2_options_cns = -x ava-ont -t 30 -k17 -w17, read_cutoff = 1k, seed_cutoff = 15k), NECAT [16] (GENOME_SIZE = 3,900,000,000, other parameters were default) and WTDBG2 [22] (-p 19 -AS 2 -s 0.05 -L 5000) were used to assemble long reads that passed quality control, respectively. The two software Nextdenovo and NECAT will perform self-correction on ONT long reads firstly and generate two corrected reads sets, namely ONT-1-correct and ONT-2-correct.

### Assembly polishing

The three initial assemblies were polished using NextPolish [15] in best mode with long reads and short reads, produced ONT-nextdenovo assembly, ONT-necat assembly, and ONT-wtdbg assembly. Additionally, the initial assembly using Nextdenvo was regarded as ONT-nextdenovo-0, and we performed an extra polishing for the ONT-nextdenovo assembly using CCS reads, generating the ONT-nextdenovo-2 assembly.

### Assembly evaluation and reads mapping

Assemblies were evaluated using BUSCO [23] and read mapping. BUSCO analysis was performed for the three polished assemblies. For reads mapping, bwa software was used for short reads and minimap2 for long reads. The long ONT-raw reads and corrected reads (ONT-1-correct, ONT-2-correct, and CCS read) were mapped to all ONT-based assemblies, together with CCS-hifiasm assembly. At the same time, short reads (NGS, CCS2short, ONT-1-correct2short) were also compared to these assemblies. Next, the coverage was counted by bedtools [24] (v2.28.0) with command “bedtools genomecov -ibam sort.bam -max 200”.

### Heterozygosity estimation

CCS Reads were first mapped to CCS-hifiasm assembly and secondary or supplementary mapping was filtered using “samtools view -F0 × 900”. Then we used “samtools mpileup” to pileup the sorted alignment in bam format. According to the pileup file, we filtered positions with depths less than five and calculated the supporting reads numbers and frequencies for non-reference alleles. For positions with allele frequency (AF) ≥ 0.25 and ≤ 0.75, we classified them as heterozygous sites and detected a total of 7.38 million heterozygous sites, i.e. genomic heterozygosity of 0.22%.

### Sequence error rate

Based on the bam format sorted alignment using ONT-raw reads, we calculated the sequence error rate on ONT-nextdenovo assembly. First, split the assembly into 100 bp bins, and calculate the number of read bases and the number of mismatched bases on the alignment for each bin. Then the ratio of the number of mismatched bases to the total number of bases is regarded to be the sequencing error rate of the mismatch type. Similarly, for the sequencing error rate of the gap type, we calculate the total length of the gap introduced by the comparison of the read in the bin and then divide it by the total number of bases calculated above to get the gap type sequencing error rate.

## Results

### The high proportion of UCR in ONT-based assemblies of VDL

We assembled the genome of a medicinal plant used in traditional Chinese medicine, *Veratrum dahuricum* (Turcz.) O. Loes in the family Melanthiaceae, using 192 Gb long-reads with Oxford nanopore sequencing technology (ONT). Based on the 49× data (calculated using the genome size of 3.93 Gb estimated from the flow cytometry), we got a 4.28 Gb assembly with an N50 of 5.64 Mb using the software Nextdenovo [21] (Table 1). 135 Gb Illumina short reads were mapped against the assembly to estimate genome integrity, we noticed that 24.3% of the ONT-nextdenovo assembly could not be covered by any NGS reads. To exclude the effect of the assembler, we applied another assembler using the correct-then-assemble strategy (NECAT [16]) and one non-error-correction assembler (Wtdbg2 [22]) for the initial assembling. The UCR proportion that could not be covered by NGS reads in ONT-necat assembly and ONT-wtdbg2 assembly reached 23.7% and 50.85%, respectively, indicating a large part of the ONT assemblies are UCR no matter which of the three assemblers was used.

**Table 1:** Summary of VDL genome assemblies and mapping by NGS reads

Assembly	CCS-hifiasm	ONT-nextdenovo	ONT-necat	ONT-wtdbg
Size (Gb)	3.55	4.28	3.75	3.45
N50 (Mb)	2.22	5.64	1.20	0.05
Complete BUSCOs (%)	92.63	90.09	87.92	77.51
NGS mapped rate (%)	99.60	99.60	99.52	99.29
NGS properly paired (%)	96.17	91.44	90.36	85.03
Depth (X)	36.62	30.25	34.36	35.52
Coverage (%)	99.53	75.70	76.31	49.15
Coverage % (≥ 5×)	98.26	69.92	71.12	45.60
Coverage % (≥ 10×)	93.50	61.99	64.11	40.01
Coverage % (≥ 20×)	72.31	42.18	45.90	33.32

### High proportions of UCR are common for polyploid genomes assembled with long reads of high error rates

**Fig. 1:**
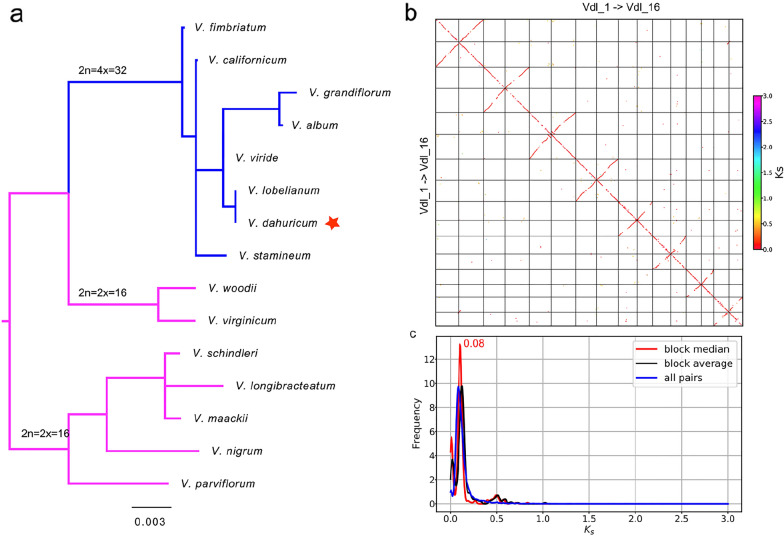
Allotetraploid inference of VDL. **a** Phylogenetic tree of *Veratrum* based on the chloroplast trnL–trnF gene spacer sequence. The data set (trnL–trnF) of 15 veratrum plants was used to build a representative family-level tree. Nucleotide sequences alignment was made using muscle software and the best tree was generated by the command “raxml-ng -msa Veratrum.trnL–trnF.fa.muscle --msa-format FASTA --data-type DNA --all --model GTR + G --threads 1 --bs-trees 100 –redo”. The phylogenetic tree is consistent with the tree constructed by Pellicer, et al. [25], and VDL is located in “2n = 4x = 32” clade, suggesting tetraploid. **b** Dot-plot of VDL orthologs, collinearity analysis of the CCS-hifiasm assembly was conducted using WGDI pipeline [28]. **c** The synonymous substitutions (*K*s) frequency density distributions of orthologs, the *K*s peak was detected to be 0.08

Phylogenetic analysis based on the chloroplast trnL–trnF gene spacer of VDL showed that VDL was in the “2n = 4x = 32” clade [25] on the phylogeny of the *Veratrum* genus (Fig. 1a), suggesting VDL was a tetraploid species, which may be more challenging for genome sequencing and assembly. A recent study reported that subgenome sequence divergence (measured in synonymous substitutions per synonymous site, *K*s) since allopolyploidization of six allopolyploid angiosperms ranged from 0.026 to 0.105 [26], while sequence divergence between allelic chromosomes of autotetraploid alfalfa is much lower, peaked at ~ 0.01 [27]. We applied the WGDI pipeline (whole-genome duplication identification v0.4.7 [28]) to perform collinearity analysis of the CCS-hifiasm assembly [20], the most recent WGD event was the polyploidization of VDL and was consistent with the peak of *K*s at 0.08 (Fig. 1b, c), which was located in the subgenome sequence divergence range of the allopolyploid angiosperms, suggesting the tetraploid species probably to be an allopolyploid.

To check the performance of third generation sequencing on other allopolyploid species, we compared the NGS coverages of the two published allotetraploid genomes assembled using ONT reads. For species *Brassica carinata *[29] and *Miscanthus lutarioriparius *[30], 13.1% and 5.4% of the non-Ns genomic regions could not be covered by NGS reads, respectively. And we also checked several genomes assembled with long reads of Pacbio single-molecule real-time (SMRT) platform (not in the high fidelity CCS mode), finding that 20.4%, 1.8%, 9.8%, 26.9%, 3.7%, and 6.6% of non-Ns genomic regions of *Arachis monticola *[31], *Arachis hypogaea *[32], *Brassica juncea* var Varuna [33], *Brassica juncea* var. timuda [34], *Gossypium barbadense* and *Gossypium hirsutum *[35] could not be covered by NGS reads (Additional file 1: Table S1). The results indicate that a high percentage of UCR can be found in many allotetraploid genomes assembled using both ONT and SMRT reads.

### UCRs are mostly a result of sequence errors in the assembly

**Fig. 2:**
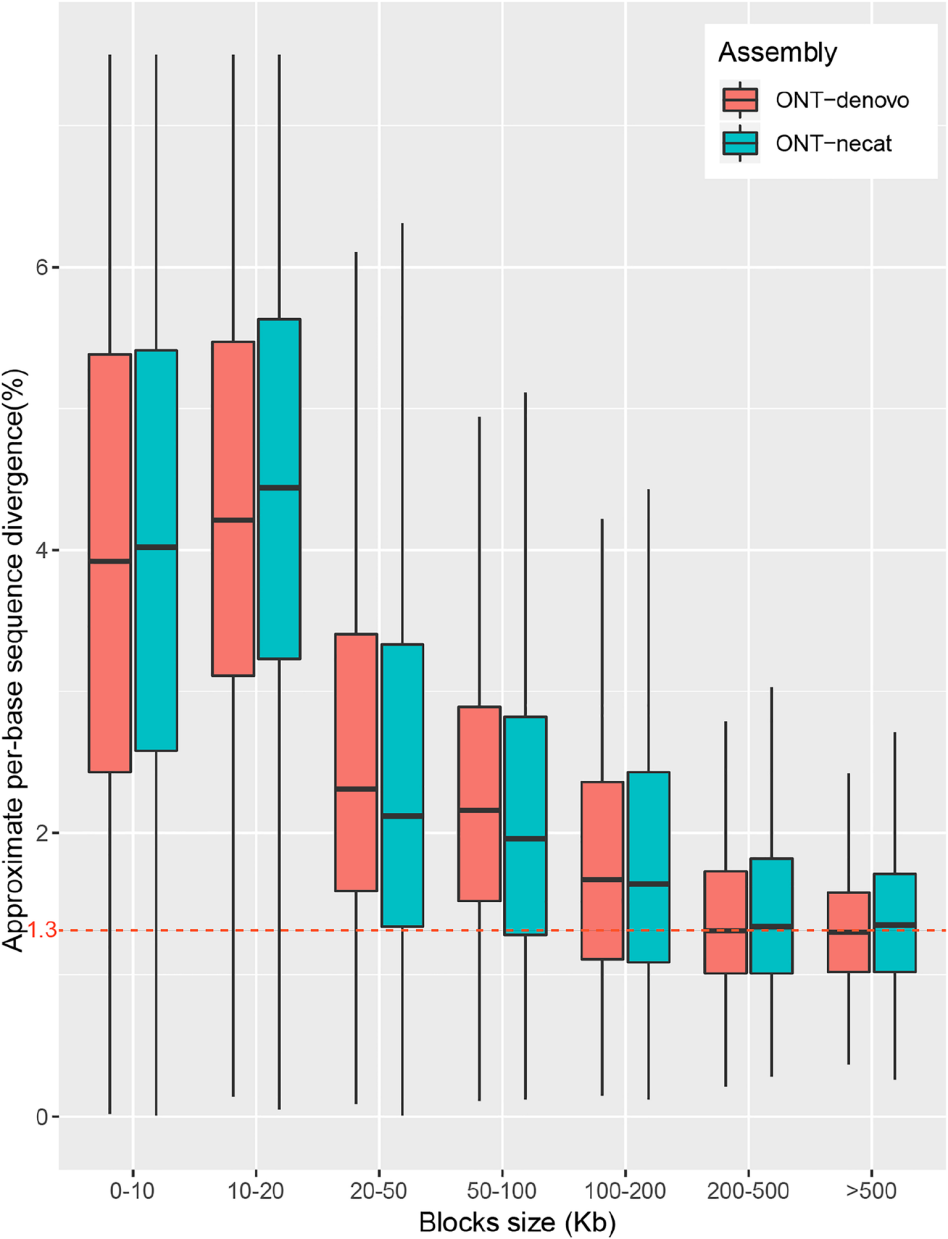
Nucleic acid alignment between ONT assemblies and CCS-hifiasm assembly. Two ONT-based assemblies were mapped to chromosome-level CCS-hifiasm assembly using minimap2, and the approximate per-base sequence divergence of each block was extracted from alignments. Blocks were grouped according to size

**Fig. 3:**
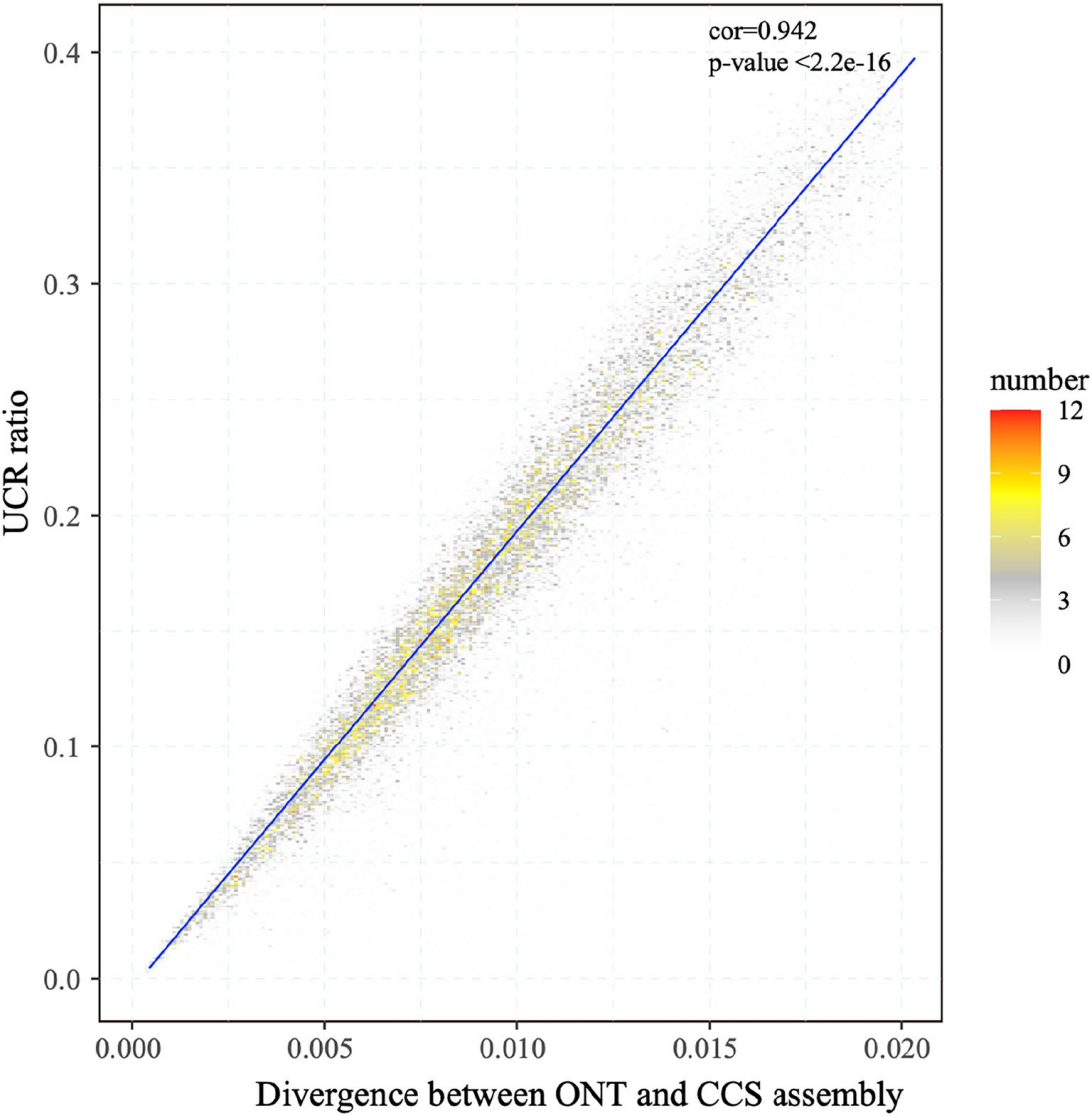
The UCR ratio and divergence between ONT assembly and CCS-hifiasm assembly. We cut the (> 200 kb) blocks between ONT-nextdenovo assembly and CCS-hifiasm assembly into 100 kb bins and counted the divergence between ONT-nextdenovo assembly and ONT-hifiasm assembly, finding that the divergence was positively associated with UCR ratio (cor = 0.942, p-value < 2.2e–16)

UCR is probably the result of sequence errors in the assembly or from the genome sequences that cannot be sequenced by NGS due to the sequencing bias of NGS. To clarify the nature of UCR, we first aligned the sequences of the ONT-nextdenovo assembly with the chromosomal CCS-hifiasm assembly which was often regarded as the standard sequence due to the much higher accuracy of the CCS HiFi reads [20]. We found that 97.57% of the CCS-hifiasm assembly is covered by alignment blocks longer than 10 kb (Additional file 1: Table S2) while 91.73% of the ONT-nextdenovo assembly is covered. These results indicate that the ONT-nextdenovo assembly and CCS-hifiasm assembly shared most of the sequences and there is only slightly more proportion (8.27%) of ONT-specific sequences than the proportion (2.43%) of CCS-specific sequences. Thus, most of the UCR can be covered by CCS assembly, suggesting most of the UCR may contain a high density of errors that prevent proper alignment of the short NGS reads. By comparing the sequence difference in the alignment blocks between the two assemblies, we found that the difference between the two assembled sequences was above 1.3% (Fig. 2). As the heterozygosity of VDL is estimated to be only 0.22%, which is calculated using CCS reads, those sequence differences are mostly attributed to sequence errors instead of heterozygosity. For the 4979 blocks (with size ≥ 200 kb) (Additional file 1: Table S2), we cut them into 100 kb bins and counted the difference between the CCS assembly and the ONT-nextdenovo assembly. We found that the difference ratio was significantly positively associated with the UCR ratio (cor = 0.942, p-value < 2.2e–16, Fig. 3), suggesting that the UCR may be caused by sequence errors in the ONT assembly.

**Fig. 4:**
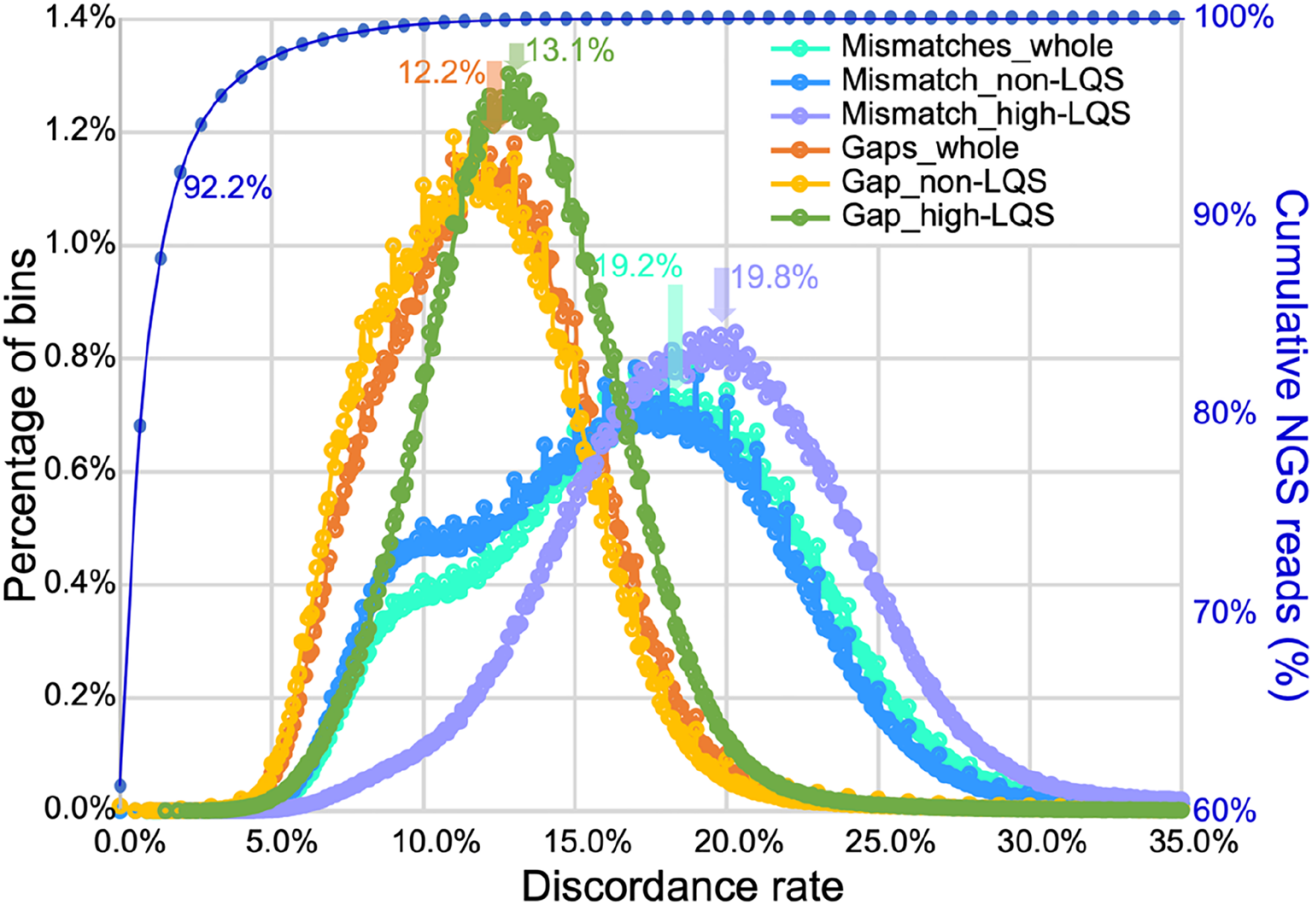
Distributions of discordance rate of ONT and NGS reads. The discordance between ONT-raw reads and ONT-nextdenovo assembly was calculated in 100 bp bins, bins with UCR length > 90% were regarded as high UCR. Both distributions of mismatch and gaps sequencing error in high UCR are higher than that of the whole genome. The blue cumulative line represents the cumulative distribution of NGS reads with mismatch rate, 92.2% of mapped NGS reads have a mismatch rate of ≤ 2%, and the average genome-wide mismatch rate is 0.68%

**Fig. 5:**
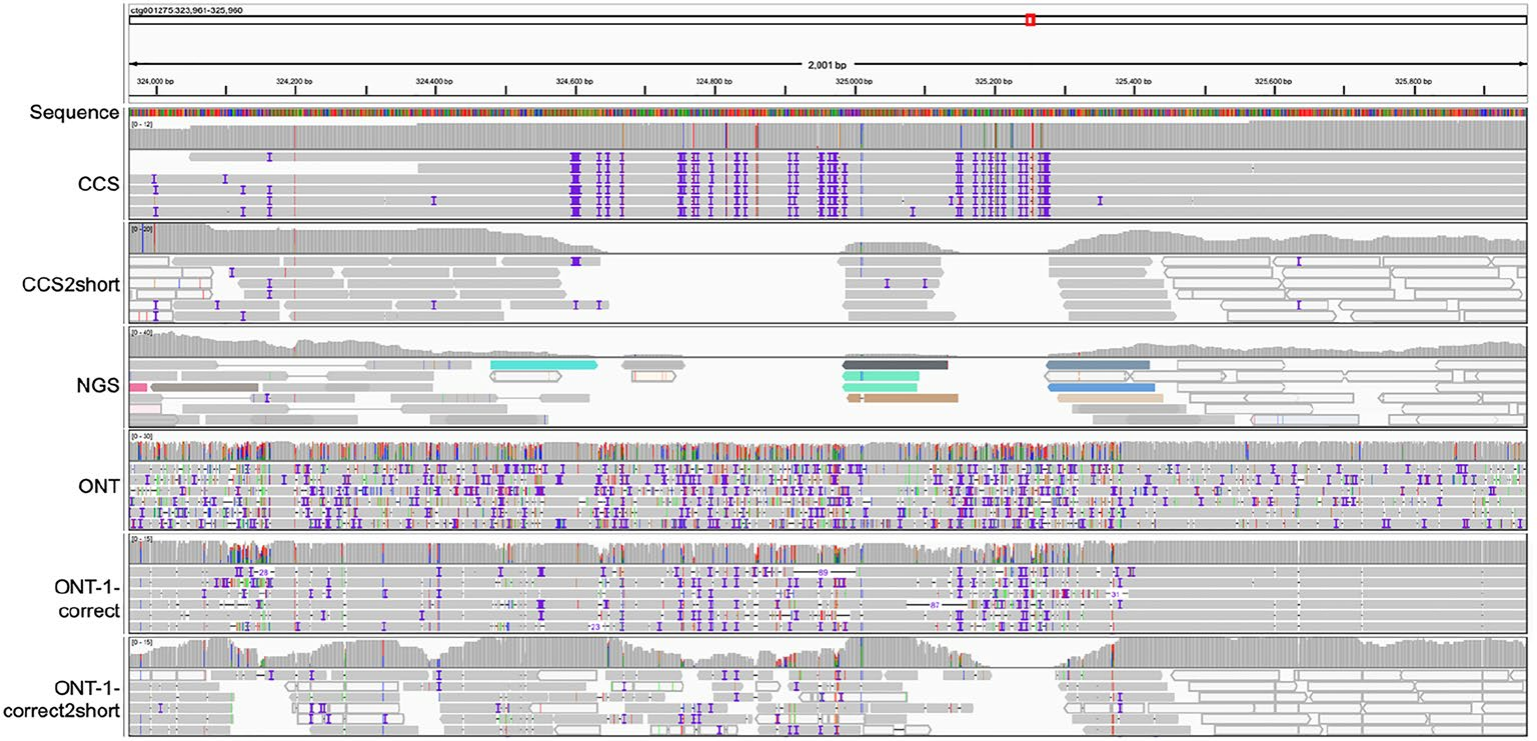
An example of sequence reads mapped to ONT-nextdenovo assembly. The 20 kb region of ctg001275 of ONT-nextdenovo assembly was used to show the reads mapping by CCS, CCS2short, NGS, ONT, ONT-1-correct, and ONT-1-correct2short reads. The mismatch of long reads is higher in areas where short reads are not covered

We further mapped the ONT-raw reads to the ONT-nextdenovo assembly and analyzed the sequence difference between them. The aligned base proportion of the ONT-raw reads is only 59.25%, and those aligned 114.06 Gb is equivalent to a genome depth of 26.67× (Additional file 1: Table S3). The average depth of ONT-raw reads in the UCR is 22.63×, about 4× (15.15% of 26.67×) lower than that of the whole ONT-nextdenovo assembly. In those aligned reads, we found that the mismatch ratio and gap ratio of ONT-raw reads that mapped to the UCR was 20.45% and 9.39%, respectively, which were 1.24 times that of the whole genome (16.48% and 7.58%), suggesting that ONT-raw reads mapped to UCR are biased towards higher sequencing error rates or higher errors in those regions of the assembly. In addition to the overall mismatch and gap ratio of all UCR, we also compared the distribution mismatch and gap ratio over sliding windows of high-UCR and non-UCR regions in the genome. We cut the ONT-nextdenovo assembly into 100 bp bins and counted the mismatches and gaps in each bin. The results of distributions showed that the peaks of mismatches and gaps reached 19.2% and 12.2%, respectively (Fig. 4). We categorized 8,225,670 bins (822.5 Mb in total) containing 90% of sequences that cannot be covered by NGS reads and regarded these bins as high-UCR and other bins as non-UCR. It is obvious that at the position of 10% mismatch, the mismatch frequency distribution of non-UCR and whole-genome has a secondary peak, which does not exist in the mismatch distribution of high-UCR (Fig. 4). In addition, the peaks of the discordance rate distributions for these high UCR shifted to the right, indicating higher discordance rates in the high UCR regions. When we mapped the CCS reads to ONT-nextdenovo assembly, we also noticed that higher discordance rate in the UCR than that in non-UCR (Fig. 5).

In summary, we compared the genomes assembled based on the two sequencing strategies and found a higher rate of sequence difference between the two genomes in the UCR than that in other parts of the assemblies. We also found that ONT-raw reads mapped to UCR have higher discordance rates. These results suggest that UCR in ONT assembly of VDL are mostly sequences with high-frequency errors in the assembly instead of true genome sequences missed by NGS. We further compare the raw reads and reads after self-correction to clarify the origin of the errors in the UCR.

### Origin of sequence errors in UCR

To further characterize the origin of errors in the UCR of assembly, we analyzed the errors in ONT-raw reads, self-corrected ONT reads, ONT assembly without polish, and the final ONT assembly by mapping them with CCS reads or CCS assembly.

First, we mapped the ONT-raw reads to the CCS-hifiasm assembly to analyze the errors in raw reads. Totally, we found 17.5% mismatch and 7.5% gaps (Additional file 1: Table S3). To compare the reads in UCR regions and other genomic areas, we mapped the UCR in ONT-nextdenovo assembly to the CCS-hifiasm assembly and categorized the ONT reads mapped to the corresponding region of UCR in CCS assembly as the UCR reads. In the UCR reads, there are 20.55% mismatches and 8.44% gaps while in non-UCR reads, there are 16.79% mismatches and 7.41% gaps, respectively (Additional file 1: Table S3). Mapping of ONT-raw reads in UCR showed a higher density of mismatches and gaps, suggesting higher sequencing error rates in raw reads of the UCR, consistent with the previous results using ONT-nextdenovo assembly as reference.

Then, we mapped the ONT-1-correct reads to the CCS-hifiasm assembly to analyze the errors in self-corrected reads. A similar analysis with the raw reads was carried out. The results showed that self-corrected reads have much fewer mismatches (7.79–8.67%) and gaps (3.04–3.3%) than raw reads both in non-UCR and UCR regions. Self-correction has reduced mismatches by 57.00% and gaps by 61.82% compared with raw reads in the whole genome while the reduced mismatch and gaps percentages are 53.77% and 54.01% in UCR (Additional file 1: Table S4). The effect of self-correction on removing sequencing errors is weaker in UCR than that in non-UCR regions. In addition, we also found 1.06% mismatches and 0.57% gaps that only in self-corrected reads but not in the raw reads, suggesting that there are also errors introduced by self-corrections. And in UCR, the mismatch errors (1.39%) introduced by self-correction are more abundant than that (0.98%) in non-UCR.

**Fig. 6:**
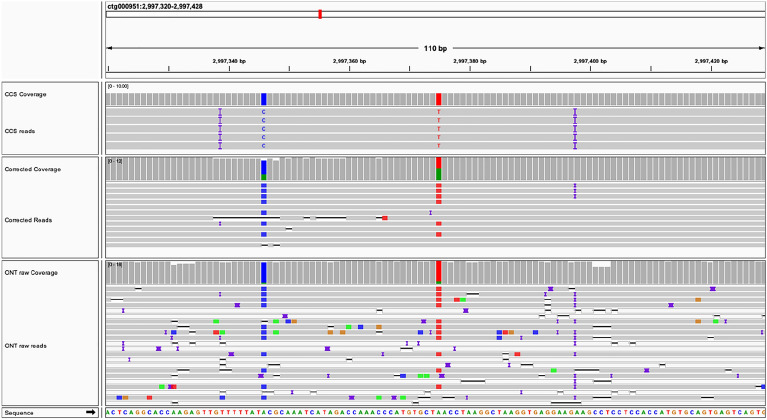
Complete discordances covered by long reads. Two complete discordances were detected using CCS reads. Correspondingly, in ONT reads and Corrected reads, the discordance rates (coverage tracks) were close to 100% and 50%, respectively. It suggests that the genotypes of ONT raw reads were consistent with that of CCS reads, but the error-correction process introduces errors, resulting in nearly half of the genotypes of the corrected reads being different from CCS reads. Multiple reads are secondary mapping (blank strips) in the ONT reads alignments, and their primary alignments were in other homologous regions, which may interfere with the error-correction process. The blue, red, green, and orange blocks represent “C”, “T”, “A”, and “G” genotypes, respectively. Gray and blank strips represent primary alignment and secondary alignment, respectively

Finally, we mapped CCS reads to the ONT-nextdenovo-0 assembly without polish to analyze the errors in the initial ONT assembly and to the ONT-nextdenovo assembly after polish to analyze the errors in the final assembly. The mismatches and gaps are much rarer than that in ONT reads. But the fold difference between UCR and non-UCR increased to 2.91 for mismatches and 2.14 for gaps in the unpolished assembly. After polishing, the fold difference between UCR and non-UCR in the final assembly increased to 17.67 and 15.35, respectively, suggesting polishing reduced a large number of sequence errors in non-UCR regions. We also found that in UCR, 54.92% of mismatches and 61.60% gaps were complete discordances in all reads at the sites, indicating these sites of different genotypes with the assembly that constitute 5.39% of the UCR sequences, which is much higher than the mean mismatch rate (0.68%) of NGS reads (Fig. 4), may play an important role in the missing of NGS reads aligned to these regions (Additional file 1: Table S5). By comparing CCS reads and ONT reads before and after error-correction, we can find the sites with complete discordances, where the genotypes of ONT raw reads and CCS reads are the same, and both are homozygous, but misalignment of reads introduces confusion to error-correction, resulting in heterozygous genotypes after error-correction (Fig. 6).

Additionally, we found that CCS reads can be added to fix most of the errors in the UCR. When polished with CCS reads, the coverage of the ONT-nextdenovo-2 assembly by NGS reads increased to 95.21%, which is about the same level as the CCS-hifiasm assembly (Additional file 1: Table S6).

To sum up, the sequence errors have been reduced along each of the three steps of ONT assembly. But the errors are more efficiently reduced in non-UCR regions. The UCR in the ONT-nextdenovo assembly is a result of errors from sequencing, self-correcting, and polishing.

## Discussion

**Fig. 7:**
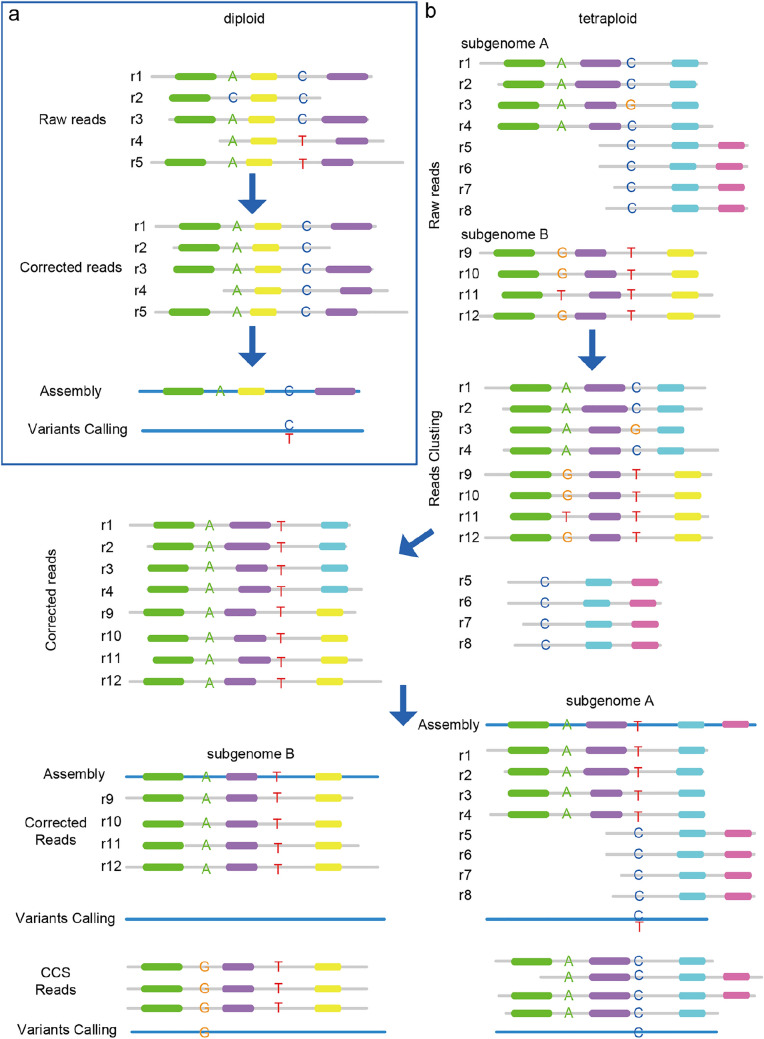
A pattern of ONT reads self-correction. **a** For diploid, the homozygous base ‘A’ and the heterozygous base ‘C/T’ were corrected to be ‘A’ and ‘C’, respectively. Colorful blocks stand for conserved regions. **b** for tetraploid, in subgenome-A, reads r3 was sequenced “C->G” in error, and reads r11 of subgenome-B was “G->T”. As the existence of conserved regions (green and purple), reads r1–4, and r9–12 were clustered to perform error correction, resulting in two homozygous SNPs for the subgenomes using CCS reads and one heterozygous SNP using corrected ONT reads

As we can see that UCR frequently occurred in polyploid genomes and observe more than 5% complete discordances in UCR sequences. Here, we propose a possible model to explain the causes of UCR. Namely, during the self-correction of ONT reads, the similarity between the two subgenomes of the VDL leads to the chimeric cluster of reads. Those chimeric clusters are corrected according to one sequence in the cluster, resulting in correction errors. Those corrected reads then lead to regions with a high proportion of errors that prevent proper mapping of NGS reads. Therefore, we inferred a self-correction pattern of ONT reads that led to different genotypes (Fig. 7). Unlike diploid species, whose sequencing errors of homozygous bases can be corrected easily, and heterozygous may cause genotype loss (Fig. 7a), the situation of tetraploid species is more complicated. In tetraploid, ONT-raw reads of r1–r8 were obtained from the subgenome-A and r9–r12 obtained from subgenome-B. Reads r1–r4, and r9–r12 are homologous on the left sides (green and purple blocks) and diverged on the right sides (blue and yellow blocks). Reads r5–r8 are from another adjacent region of subgenome-A, which overlaps with r1–r4, but does not overlap with r9–r12. During all versus all alignment, r1–r4, and r9–r12 will be clustered following conserved regions (green and blue blocks). Two “homozygous” sites (“A”, “C” of subgenome-A and “G”, “T” of subgenome-B) will constitute heterozygous sites. Due to sequencing error, reads r3 were sequenced “C->G” in error, and reads r11 of subgenome-B was “G->T”, making “A” and “T” the major allele. Hence, like diploid, r1–r4, and r9–r12 were corrected to be “A” and “T” at the two sites. When CCS reads are mapped to assembled subgenome-A and subgenome-B, both subgenomes have a homozygous SNP. But for corrected ONT reads mapping to subgenome-A, r5-r8 with the correct genotype “C” will introduce a heterozygous SNP (Fig. 7b).

## Conclusions

In summary, based on a comparative analysis of multiple assemblies and reads from the same plant, we analyzed the errors that contributed to the high proportion of UCR. A possible model was proposed to explain the formation of UCR in polyploid genomes. The model predicts that ONT reads or any other reads with high error rates are not suitable for self-correction and assembling of polyploid genomes. We suggest polishing with high-precision CCS reads to efficiently correct those errors in ONT assembly.

## Supplementary Information

**Additional file 1: Table S1.** Published allotetraploid genomes covered by short reads sequencing. **Table S2.** Blocks between nextdenovo assembly and chromosome-level hifiasm assembly. **Tables S3.** Discordance error in long reads mapped to different area of assemblies. **Table S4.** Postions with mismatch and gap of CCS-hifiasm assembly. **Table S5.** Discordance of CCS reads mapped to ONT-nextdenovo assembly. **Table S6.** Reads mapping and genome coverage

## Data Availability

Raw data and assembled genome in the chromosomal level of *Veratrum dahuricum* have been deposited at National Genomic Data Center (https://bigd.big.ac.cn/bioproject/) under the accession number PRJCA005207. For ONT-based assemblies or more data requirements, please contact jingcai@nwpu.edu.cn.

## Abbreviations

CCS: Circular consensus sequencing
CCS2short: The CCS reads were cut into non-overlap 150 bp short reads
CCS-hifiasm: The hifiasm assembly using CCS reads
NGS: Next Generation Sequencing
ONT: Oxford nanopore sequencing technology
ONT-nextdenovo-0: The initial nextdenovo assembly using ONT reads without polishing
ONT-nextdenovo: The nextdenovo assembly using ONT reads with the best mode polishing
ONT-nextdenovo-2: The ONT-nextdenovo assembly with an extra polish using CCS reads
ONT-necat: The necat assembly using ONT reads with the best mode polishing
ONT-raw: Un-corrected ONT reads
ONT-wtdbg: The wtdbg assembly using ONT reads with the best mode polishing
ONT-1-correct: Corrected ONT reads using Nextdenovo
ONT-1-correct2short: The ONT-1-correct reads were cut into non-overlap 150 bp short reads
ONT-2-correct: Corrected ONT reads using NECAT
SMRT: Single Molecule, Real-Time
UCR: Un-Covered Region by NGS reads
VDL: *Veratrum dahuricum* (Turcz.) O. Loes
